# Supplementary material for: Additive Manufacturing of an Extended-Release Tablet of Tacrolimus
Source: Materials (Basel). 2023 Jul 10;16(14):4927. doi: 10.3390/ma16144927 (PMC10381679; doi:10.3390/ma16144927)
Supplement: Supplementary file 1 [file materials-16-04927-s001.zip › materials-1852515-supplementary.pdf]

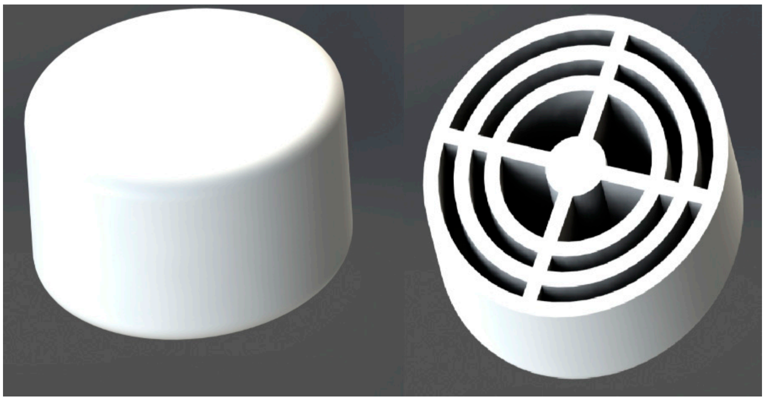

Figure S1. Exterior (left) and interior (right) layout of the pill shell designed by Solid works.

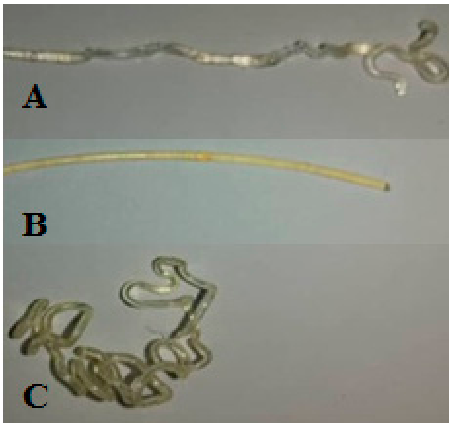

Figure S2. From top to bottom: A) brittle filament; B) extrudable and printable filament; C) extrudable but not printable filament.

Table S1. Different formulations of HPMC gel for printing by PAM

Formulations and amounts	HPMC polymer	Solvent volume (mL)	Extrudability	Printability
F1	10	200	×	×
F2	20	200	✓	✓
F3	30	200	✓	×

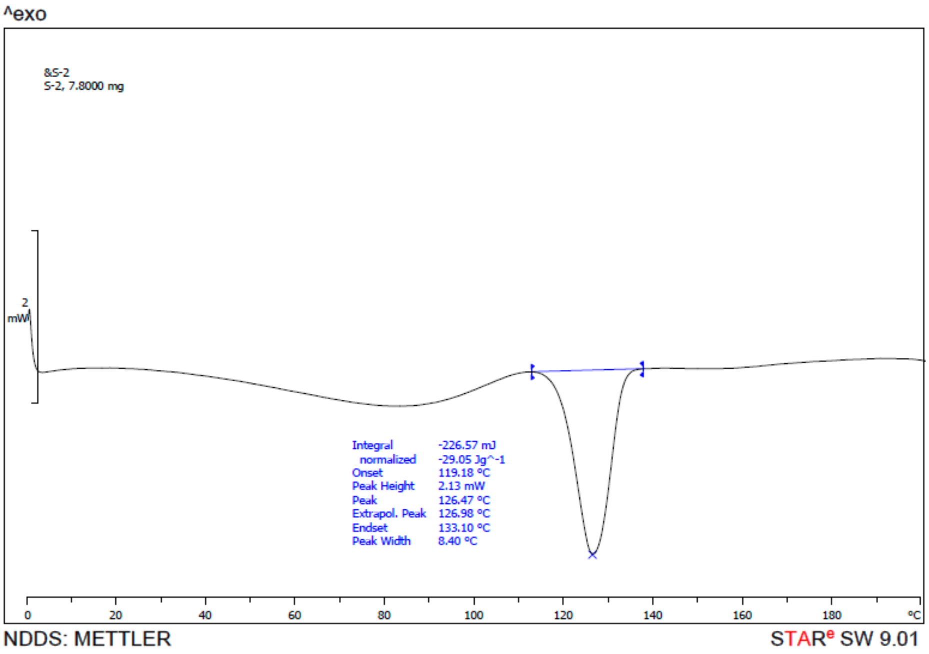

Figure S3. Diagram of DSC thermal analysis of Tacrolimus

Table S2. Assay determination of 3D printed tablet of Tacrolimus

	**Assay**		
	Area	Concentration (ug/ml)	% Assay
**Injection1**	227.00	19.82	99.10
**Injection2**	229.40	20.02	100.12
**Injection3**	223.00	19.48	97.40
**Average**	226.47	19.77	**98.87**
**STD**	3.23	0.28	1.38
**RSD**	1.43	1.39	1.39

Table S3. Content uniformity assay of 3D printed pills

Tablet no.	Area	concentration (ug/ml)	%
Tablet 1	223	19.39	97.0
Tablet 2	237	20.67	103.4
Tablet 3	230.7	20.14	100.7
Tablet 4	235	20.50	102.5
Tablet 5	226	19.73	98.7
Tablet 6	234	20.42	102a.1
Tablet 7	219	19.14	95.7
Tablet 8	213	18.63	93.1
Tablet 9	220	19.22	96.1
Tablet 10	241	21.01	105.1
Average(Ẍ)	227.8	19.89	99.4
STD	8.91	0.76	3.8
RSD (S)	3.91	3.82	3.8
T=Target		99	
Average(Ẍ)		99.4	
S		3.8	
Acceptance Value	KS	**9.12**	Limit <15

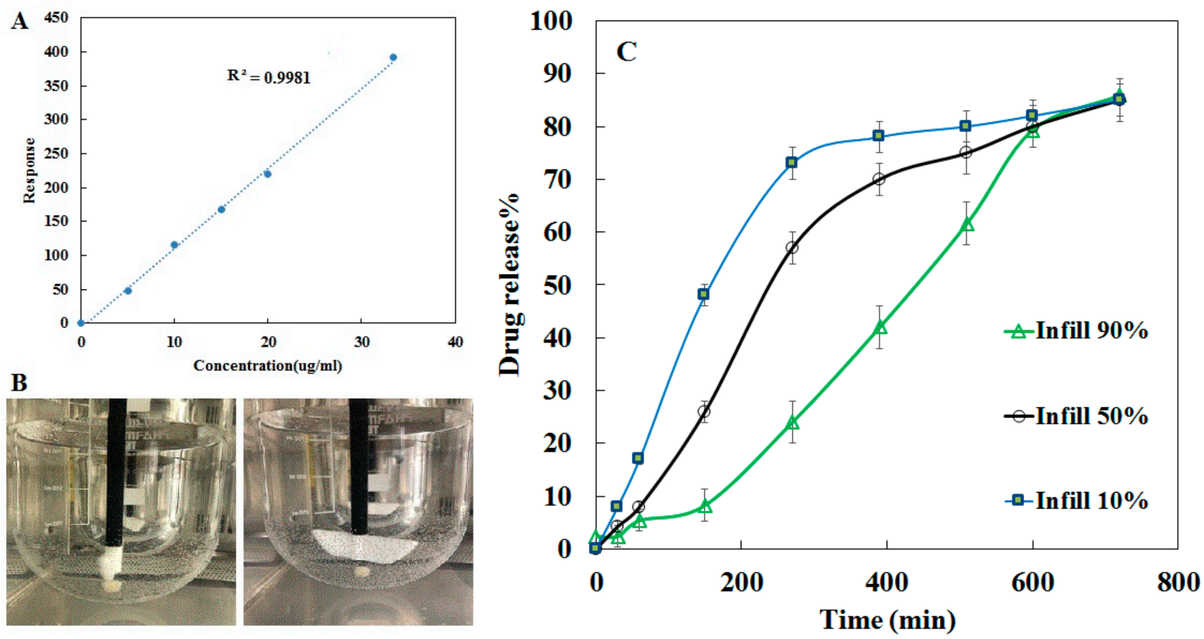

Figure S4. Calibration curve of tacrolimus standards for determination of dissolution samples (A). Photos taken from dissolutions 3D printed pill after 2h (Left side) and 8h (right side) (B). Dissolution profiles in the different infill % of the 3D printed tablet..

Table S4. Weight variation of 3D printed tablet of Tacrolimus**.**

Row	Weight (mg)
**1**	591
**2**	568
**3**	597
**4**	582
**5**	570
**6**	597
**7**	560
**8**	596
**9**	590
**10**	599
**Mean**	585
**STD**	14.20
**RSD**	**2.43**

Table S5. The results of tablet friability for 3D printed tablet of tacrolimus

**Friability Test**	
**Row**	Weight (mg)
**Mean W0**	589.8
**Mean Wf**	589.1
**% Friability**	**0.12**
**%AV**	1.00
